# Towards High Accuracy Reflectometry for Extreme-Ultraviolet Lithography

**DOI:** 10.6028/jres.108.025

**Published:** 2003-08-01

**Authors:** Charles Tarrio, Steven Grantham, Matthew B. Squires, Robert E. Vest, Thomas B. Lucatorto

**Affiliations:** National Institute of Standards and Technology, Gaithersburg, MD, 20899-8410 USA

**Keywords:** extreme ultraviolet, lithography, metrology, reflectometry, synchrotron radiation

## Abstract

Currently the most demanding application of extreme ultraviolet optics is connected with the development of extreme ultraviolet lithography. Not only does each of the Mo/Si multilayer extreme-ultraviolet stepper mirrors require the highest attainable reflectivity at 13 nm (nearly 70 %), but the central wavelength of the reflectivity of these mirrors must be measured with a wavelength repeatability of 0.001 nm and the peak reflectivity of the reflective masks with a repeatability of 0.12 %. We report on two upgrades of our NIST/DARPA Reflectometry Facility that have given us the ability to achieve 0.1 % repeatability and 0.3 % absolute uncertainty in our reflectivity measurements. A third upgrade, a monochromator with thermal and mechanical stability for improved wavelength repeatability, is currently in the design phase.

## 1. Introduction

In an extreme ultraviolet lithography (EUVL) stepper, a reflective mask patterned with an absorber is imaged at the wafer plane by a series of multilayer-coated mirrors. Current systems use Mo/Si coatings working at a wavelength of 13.4 nm. [1] The multilayers are periodic structures that reflect a bandpass of several percent around a wavelength determined by the period and angle of incidence. In order to achieve 70 nm printing resolution, the optical surfaces must be nearly perfect, and the deposition of the multilayer coatings must add less than 0.25 nm rms of figure error, leading to strict requirements on thickness control over the entire optical surface. These metrology requirements are dictated by the accuracy required for the final, coated optics. The metrology requirements for the Beta-tool stepper have been clearly stated by Gullikson et al. [2]: wavelength repeatability, 0.01 %; wavelength uncertainty, 0.03 %; positional uncertainty, 0.04° in angle and 0.5 mm in linear displacement; peak reflectance repeatability, 0.12 % and uncertainty, 0.5 %.

The NIST/DARPA EUV Reflectometry Facility consists of three parts: the Synchrotron Ultraviolet Radiation Facility (SURF III) [3] storage ring, which provides continuum EUV radiation; the monochromator; and the reflectometer sample chamber. The monochromator is described in detail in Ref. [4]. Briefly, a Ni-coated toroidal mirror collects 20 mrad horizontally and the full vertical extent of the emission from SURF III and focuses the radiation onto an entrance slit. The radiation is deflected and refocused by a second toroid and is incident on one of two gratings: a 600 mm^–1^ grating blazed for 20 nm, which we operate between 8 nm and 40 nm, or a 1500 mm^–1^ grating blazed for 8 nm, which we use from 3 nm to 14 nm. Wavelength is scanned by a plane Au-coated mirror that rotates and translates, reflecting the selected wavelength to the exit slit, which resides about 55 cm within the large sample chamber.

The NIST/DARPA EUV Reflectometry Facility is unique in two ways. First, it is the only such facility in the world capable of handling the large optics encountered in present designs of EUV steppers. The instrument is currently configured to handle optics as large as 35 cm in diameter and 50 kg in mass. The recently commissioned Engineering Test Stand (ETS) at Sandia National Laboratory, built in collaboration with Lawrence Livermore and Lawrence Berkeley National Laboratories, presents an example illustrating the need for this capability. The ETS has an illumination system with two near-normal-incidence mirrors and two grazing reflections, and a four-mirror imaging system. The largest mirror of this system is the first condenser, or C1 optic, which has six sectors and is 27 cm in diameter and 7.5 cm thick at the edge. The NIST/DARPA reflectometer is currently the only instrument in the USA large enough to make a complete set of measurements on the intact C1 mirror. Keeping the C1 system intact during measurements is important because it reduces the time and cost associated with the removal and replacement of the sectors after coating.

**Fig. 1:**
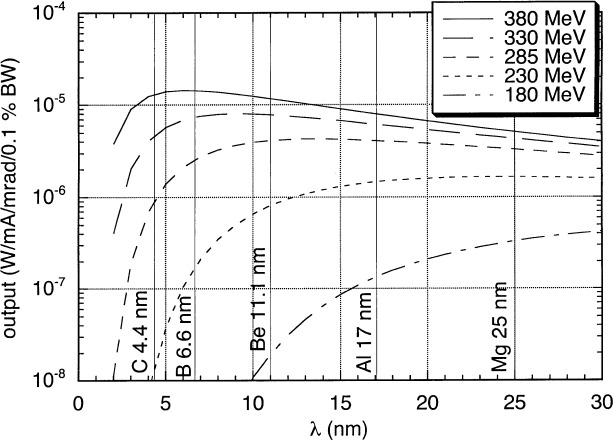
Calculated emitted power from the SURF III storage ring per horizontal milliradian of intercepted orbit, per milliampere of beam current in a 0.1 % bandwidth as a function of wavelength *λ* for various energies between 180 MeV and 380 MeV and assuming collection of the full vertical extent of the emission. The vertical lines indicate the transmission edges of various filter materials.

The second unique feature of the NIST/DARPA Facility is the tunablity of the source. SURF III is a single-magnet electron storage ring with an 83.7 cm radius. It can be operated reliably at electron beam energies from 100 MeV to 380 MeV. This allows us to adjust the peak output wavelength to minimize out-of-band radiation due to scattered and higher-order radiation. Furthermore, when used with a combination of filters and detectors, we can determine the effects of out-of-band radiation on the reflectivity measurement. Figure 1 shows the spectral intensity of the source for various energies between 180 MeV and 380 MeV. Vertical lines indicate the absorption edges of available filters—C, B, Be, Al, and Mg—which are used to reduce higher diffraction orders and long-wavelength scatter from the grating. As can clearly be seen, the ratio of in-band power at 13.4 nm to out-of-band power can be varied by orders of magnitude by varying the electron beam energy over this range.

## 2. Sample Chamber Goniometer

Samples are mounted on a 42 cm diameter platen with six-axis motion. The entire assembly rotates about a horizontal axis to set the angle of incidence *θ*. The transverse angle can also be varied between –30° and 30° to keep the reflection in the vertical plane when measuring curved samples, or to correct for wedge angles in flat samples when necessary. There are three degrees of translation: 35 cm in the horizontal direction, *x*; 10 cm in the vertical, *y*; and 5 cm of “piston” or *z* motion. The piston motion is adjusted to bring the sample surface to the center of rotation of the *θ*-axis. This adjustment is made by monitoring the reflected spot using an EUV-sensitive camera on the detector arm, and is very important in order to keep the reflected spot at the same position on the detector. The camera is a simple visible-sensitive, charge-coupled-device (CCD) camera on a small printed-circuit board in an O-ring sealed box with a phosphor-coated front screen. Finally, there is a continuous azimuthal rotation of the platen, *ϕ*. The combination of *x* and *ϕ* motions allow us to scan the entire surface of an optic up to 35 cm in diameter. The motion control is described more completely in Ref. [5].

There are also two detector axes. The primary or 2*θ*-axis is coincident with the horizontal *θ*-axis of the sample. There is also a second degree of freedom for the detector carriage that consists of motion along an arc on the detector arm which is transverse to the 2*θ* motion. This motion serves two purposes: first, it allows us to switch detectors without breaking vacuum; and second, the ±15° of motion in addition to the ±30° of sample tilt allows us to measure optics with slopes of up to 37.5° by observing reflections out of the vertical plane. Given that our beam is 96 % horizontally polarized, measurements with out-of-plane reflections must be corrected for the reduced reflectivity of p-polarized reflections. This is a small correction, though, even at 7.5°.

Most of these motions are actuated by stepper motors through UHV bellows-sealed feedthroughs. However, due to the large mass of the assembly, the *θ* motion is actuated directly through a differentially pumped rotary seal.

Initial positioning and alignment of the sample are done with the aid of several CCD cameras, which monitor the sample and exit slit within the vacuum chamber. Normal incidence is set by retroreflecting a laser that is collinear with the EUV radiation, which can determine normal to better than 0.05°. Sample edges can be located to 0.2 mm, and the resolution of the translation axes is 25 µm. The position on the detector can be kept constant to 0.2 mm by viewing the reflected beam with the EUV-sensitive camera, which is displaced from the detector by 8.0°.

This entire assembly is housed in a 2 m diameter by 3 m long vacuum chamber pumped by a dry roughing pump and two cryopumps. Vacuum of 10^–4^ Pa is achieved in about an hour, and <10^–5^ Pa overnight. The chamber can be opened to the synchrotron about 1 h after beginning the pumpdown cycle. The ultimate pressure of less than <10^–6^ Pa takes several days, but can be achieved faster by the use of available in-vacuum bakeout lamps if necessary.

## 3. Reflectivity Uncertainty

Three elements are needed to achieve high-accuracy reflectivity measurements: an accurate method of determining the flux of incident radiation at the desired wavelength, an accurate method of measuring the amount of that radiation that is reflected, and a well-characterized beam incident on the sample.

An actual reflectance measurement is done by first moving the sample out of the incident beam and placing the detector in the direct, unreflected beam to measure the amount of incident radiation. We refer to this measurement result, when normalized, as *I*_0_. The sample is then set to the appropriate position and angle, and the same detector set to provide a measurement of the reflected beam *I*_m_. The measured (as opposed to true) reflectance *R*_m_ is simply the ratio of these two results:

$$R_{m}=\frac{I_{m}}{I_{0}}.$$ (1)

*I*_0_ and *I*_m_ are normalized to compensate for the fact that the stored current in the synchrotron decays somewhat during the measurements.

Many synchrotron-based facilities normalize the measurements to the stored beam current. However, the proportionality constant connecting the photocurrents to the beam current may vary by a percent or more over an hour due to heating of optics, variations in the synchrotron beam position, etc., thus requiring frequent remeasurement of *I*_0_ to determine this proportionality constant.

**Fig. 2:**
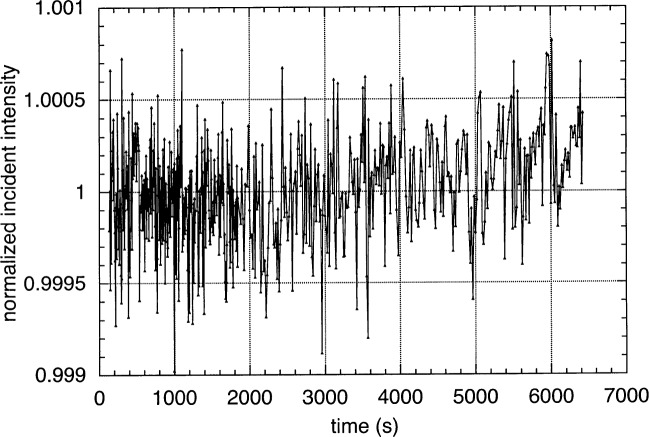
Normalized incident intensity, *I*_0_, as a function of time. Data points were measured every second toward the beginning and every ten seconds after that. The drift is 0.01 % / h and the rms noise is 0.03 %.

We have chosen a new method to provide a continuous, simultaneous, highly accurate normalization. This is accomplished by incorporating a second exit slit, identical in size to the primary exit slit, in the monochromator. There is an EUV-sensitive photodiode behind the second slit that continuously monitors the amount of radiation passing through it. This monitor slit is 2.5 mm below the primary exit slit and receives radiation that is shifted by approximately 0.25 nm to longer wavelength when the monochromator is set to 13 nm. Given the small separation between the two slits, the amount of radiation passing through the primary exit slit can be accurately tracked by the monitor. We have measured both *I*_0_ and reflectivity at a single spot on a mirror repeatedly over long periods of time and find typical drift of about 0.2 % over several hours (i.e., a beam lifetime), and a noise level of less than 0.05 %. The drift is fairly linear and further correctable to 0.05 % using interpolation. Figure 2 shows one result of the normalized *I*_0_ as a function of time over about two hours. In this case the drift is about 0.01 % / h and the rms noise is 0.03 %.

The reflectivity of a sample is measured using a single EUV-sensitive detector to measure the incident and reflected beams. This allows us to assume that the conversion from photon flux to electron current is the same for both *I*_0_ and *I*_m_ if one illuminates the same portion of the detector for each measurement to minimize the effects of detector non-uniformity. Detector placement in this system is done by viewing the reflected beam with the EUV-sensitive camera, which is displaced by a known angle from the detector. Once the detector arm has been aligned to properly place the reflected beam on the camera, the mirror angle is then changed by a known offset to place the beam onto the detector. The position of the reflected beam on the detector can be kept constant to 0.2 mm using this technique.

In order to determine the accuracy of our measurements, we must determine the amount of out-of-band radiation present in our incident beam. If we assume a linear photodetector, the true reflectivity is defined as:

$$R=\frac{I_{R}}{I_{\lambda }},$$ (2)

where *I*_R_ is the photocurrent produced by light of pure wavelength *λ* reflected from the mirror, and *I*_λ_ is the photocurrent produced by the incident light of wavelength *λ*. However, the actual measured photocurrent produced by the incident intensity is always going to have some contribution from out-of-band components that affect the measured reflectivity:

$$I_{0}=I_{\lambda }+I_{\mathrm{ex}}$$ (3a)

and

$$I_{m}=I_{R}+\Delta I_{\text{RS}},$$ (3b)

where the normalized photocurrent *I*_ex_ is the extraneous contribution from the out-of-band radiation and ∆*I*_RS_ is due to the part of the out-of-band radiation that is reflected by the mirror. Thus, the measured reflectivity is

$$R_{m}=\frac{I_{R}+\Delta I_{\text{RS}}}{I_{\lambda }+I_{\mathrm{ex}}}.$$ (4)

We can solve for these equations for *R* to get:

$$R=R_{m}\left(1+\frac{I_{\mathrm{ex}}}{I_{\lambda }}\right)-\frac{\Delta I_{\text{RS}}}{I_{\lambda }}.$$ (5)

A model described below provides a way to evaluate the out-of-band contributions in Eq. (5) by parameterizing them and determining their magnitude using measurements obtained under different conditions. The (normalized) flux coming through the exit slit when the monochromator is tuned to *λ* in the interval [*λ'*, *λ'* + d*λ'*], which we represent by the quantity *Φ* (*λ'*) d*λ'*, can be expressed as:

$$\Phi (\lambda^{\prime })d\lambda^{\prime }=T_{\lambda }(\lambda^{\prime })\cdot U(\lambda^{\prime },E)\cdot F(\lambda^{\prime })d\lambda^{\prime },$$ (6)

where *T_λ_*(*λ'*) is the throughput of the monochromator for radiation of wavelength *λ* when it is tuned to pass radiation of wavelength *λ*, *U*(*λ'*, *E*) is the vertically integrated spectral emission from SURF III with an electron energy *E* at the wavelength *λ'* in W / (nm mA mrad) given by the Schwinger equation, Eq. (6), and *F*(*λ'*) is the filter transmission.

Ideally *I_λ_* + *I*_ex_ is given by the integral over the entire wavelength range:

$$\begin{array}{c} I_{\lambda }+I_{\mathrm{ex}}=\int \Phi (\lambda^{\prime })\cdot D(\lambda^{\prime })d\lambda^{\prime }\\ =\int T_{\lambda }(\lambda^{\prime })\cdot U(\lambda^{\prime },E)\cdot D(\lambda^{\prime })\cdot F(\lambda^{\prime })d\lambda^{\prime }, \end{array}$$ (7)

where *D*(*λ'*) is the detector responsivity at wavelength *λ'*. We now make a simplifying assumption to parameterize *I*_ex_/*I_λ_*. First we note that the throughput of the monochromator could be characterized as having a main peak at the wavelength of interest, *λ*, generally smaller, higher order peaks at submultiples *λ*/*n*, and even smaller broad-band scatter that we shall label *S_λ_*(*λ'*). Thus for the case of *λ* = 13.4 nm, where we do not consider any radiation shorter than *λ*/5 we may write:

Tλ(λ′)=∑n=15Tnδ(λ′−λn)+Sλ(λ′), (8)

where the *T_n_* are some constants that represent the monochromator throughout for the nth order radiation.

The nature of *S_λ_*(*λ'*) is not known for our monochromator, thus we must make some assumption about its shape. We have used several functional forms for our scatter with similar results. We will present an analysis in which we assume that the scatter can be described by a Gaussian:

$$S_{\lambda }(\lambda^{\prime })=T_{S}e^{\frac{-{\left(\lambda^{\prime }-\lambda \right)}^{2}}{\sigma^{2}}},$$ (9)

where *λ* = 13.4 nm, *T*_S_ is a constant to be determined, and *σ* = 2 nm. This shape is similar to that found by Gullikson et al., [2] and provides a good description of our data. Assuming that the scattered background is constant over a bandpass of 10 nm about the central wavelength and zero otherwise (i.e., a “top hat”) yields similar results.

The expansion of the monochromator throughput in terms of the components of Eq. (8) allows us to write:

$$I_{\lambda }=T_{1}\cdot U(\lambda ,E)\cdot D(\lambda )\cdot F(\lambda ),$$ (10)

$$\frac{I_{n}}{I_{\lambda }}=\frac{T_{n}\cdot U(\lambda /n,E)\cdot D(\lambda /n)\cdot F(\lambda /n)}{T_{1}\cdot U(\lambda ,E)\cdot D(\lambda )\cdot F(\lambda )}\;\text{for}\;n\geq 2,$$ (11)

and

$$\begin{array}{c} \frac{I_{S}-\Delta I_{\text{RS}}}{I_{\lambda }}=\frac{T_{S}}{T_{1}\cdot U(\lambda ,E)\cdot D(\lambda )\cdot F(\lambda )}\;\\ \int (1-R(\lambda^{\prime }))\cdot e^{\frac{{\left(\lambda^{\prime }-\lambda_{0}\right)}^{2}}{\sigma^{2}}}\cdot U(\lambda^{\prime },E)\cdot D(\lambda^{\prime })\cdot F(\lambda^{\prime })d\lambda^{\prime } \end{array}$$ (12)

where *I*_S_ is the intensity due to scattering, ∆*I*_RS_ is a correction for to the amount of scattered light that is reflected by the sample, and *R*(*λ'*) is the measured reflectivity near the peak, and is extrapolated by calculating the reflectivity based on these results. The factor of [1 – *R*(*λ'*)] takes into account the ∆*I*_RS_/*I_λ_* correction in Eq. (5). The functions *U*, *D*, and *F* can all either be calculated or measured. Thus, under what seem to be reasonable assumptions, *I_λ_*_/_*_n_*/*I_λ_*, and *I*_S_/*I_λ_* are expressible in terms of some five constants *T_j_*/*T*_1_(*j* = 2, …, 5) and *T*_S_/*T*_1_.

**Fig. 3:**
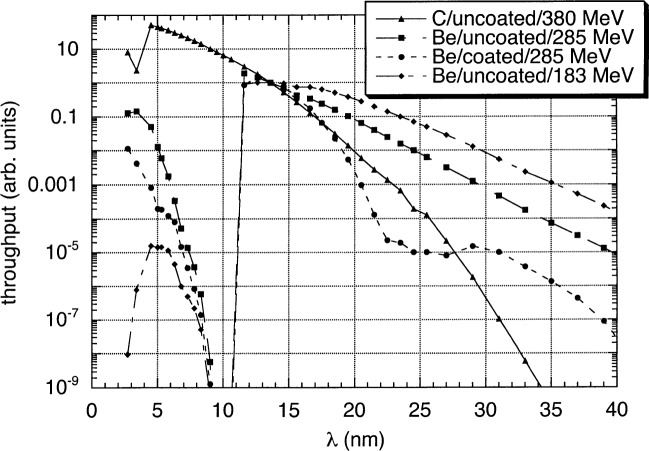
Product *U·D·F*, representing the radiant flux entering the reflectometer, as a function of wavelength *λ* for different combinations of electron beam energies, filters, and detectors.

These five unknown parameters characterizing the monochromator throughput can be determined by making measurements of a single sample under various conditions. As discussed earlier, SURF III can be reliably operated over a wide range of energies. We have made measurements at 229 MeV, 285 MeV, 331 MeV, and 380 MeV. As Fig. 1 suggests, making measurements at 285 MeV minimizes out-of-band contributions at 13 nm wavelength, while higher energies increase higher grating orders, and lower energies lead to greater long-wavelength contributions. Similarly, running with a 1 µm thick Be filter leads to a reduction of second-order radiation of over three orders of magnitude, while a 0.5 µm C filter transmits a greater fraction of 6.8 nm radiation than 13.6 nm. The C filter also rejects long wavelengths more completely than the Be. We also have both a bare EUV-sensitive photodiode and one with a ZrSi bilayer coating. The coating filters long wavelengths extremely effectively, while also offering a factor of three reduction in second-order radiation and filtering higher orders extremely effectively. Figure 3 shows the product *F*(*λ'*)·*D*(*λ'*)·*U*(*λ'*,*E*) for a few of the available combinations. This shows clearly the differences in the various operating conditions.

Table 1 presents the measured data for the measured and calculated reflectivity for a total of 15 different combinations of measurement conditions. The parameters *T_n_* and *T*_s_ are found using a least-squares fitting procedure. The average measured reflectivity is 0.6653 for the described 2 nm Gaussian model of scattered light. For comparison, we note that the result for the 10 nm top hat function bandpass is 0.6644, showing that the results are only weakly dependent on the scattered light model.

## 4. Discussion

The fitting parameters we have obtained are as follows, with the standard uncertainty in the last digit of the parameter in parentheses: *T*_5_/*T*_1_ = 0.0012(6); *T*_4_/*T*_1_ = 0.0008(8); *T*_3_/*T*_1_ = 0.0074(2); *T*_2_/*T*_1_ = 0.0295(2); *T*_S_/*T*_1_ = 0.012(5). While the uncertainties in the fourth and fifth-order terms seem large, these are very small contributions, and the uncertainties contributed to the reflectivity value due to these terms are both only 0.03 %. The uncertainty components due to the third- and second-order terms are 0.04 % and 0.03 %, while the total uncertainty is dominated by the scatter contribution of 0.3 %. This is because the scatter contribution cannot be varied as greatly using the filters. Repeated measurements of the reflectivity of a single sample, both within a day and after unmounting and remounting, indicate a statistical uncertainty contribution of 0.1 %. Total standard uncertainty with coverage factor *k* = 2, including random and systematic (type a and type b) effects, is 0.3 %.

It is apparent from the data in Table 1 that the conditions that deliberately include more out of band contributions (C filter data) lead to calculated values of the reflectivity that have a greater variation. This is most likely due to differences between the actual and assumed values of the transmission of the carbon filter. Small fluctuations in storage ring operating conditions such as beam position, noise, etc. that we are more sensitive to under sub-optimum conditions also contribute to the differences between the data sets. If we remove the data obtained using the carbon filter, but use the same parameters obtained from our fitting procedure, we obtain an average reflectivity of 0.6658 with a much tighter spread in values.

In order to compare our results for peak reflectivity with those of other facilities with higher-resolution monochromators, we must deconvolute our resolution function from our data. We can measure the resolution using a well-characterized spectral feature. We have measured the absorption of Kr in a gas cell separated from the sample chamber by a SiN window. Using this spectrum, we determined that the spectral distribution of our incident beam is well-described by two gaussians: one 0.05 nm wide, and a second with a width of 0.2 nm and an amplitude 15 % of the first. We have convoluted this function with multilayer reflectivity curves representing various peak widths, numbers of periods, and other characteristics of multilayers. Resolution correction factors vary from 1.012 for the narrowest peaks down to unity for peaks a nm or more wide.

## 5. Conclusion

We have commissioned an EUV reflectometer that is capable of measuring the largest optics of the *β*-generation of EUVL steppers. We have demonstrated both absolute uncertainty and repeatability in reflectance measurements that meet the metrological needs for this generation. Plans are underway to redesign our monochromator in order to meet the wavelength precision needs for EUVL optics.

## Figures and Tables

**Table 1:** Results of measurements and calculated reflectivity of a single test piece. The first three columns include the measurement conditions, with synchrotron beam energy, filter (1 µm Be or 0.5 µm C), and the detector used, either an uncoated photodiode or one with a Zr/Si coating. The fourth column is the measured reflectivity, and the fifth the reflectivity calculated using the method described in the text

Beam energy (MeV)	Filter	Detector	Measured reflectivity	Calculated reflectivity
380	Be	Coated	0.66106	0.66525
331	Be	Coated	0.66135	0.66520
285	Be	Coated	0.66200	0.66567
229	Be	Coated	0.66190	0.66539
380	Be	Uncoated	0.65320	0.66588
331	Be	Uncoated	0.65795	0.66634
285	Be	Uncoated	0.66065	0.66705
229	Be	Uncoated	0.66055	0.66613
380	C	Coated	0.53850	0.66577
331	C	Coated	0.56590	0.66385
285	C	Coated	0.60210	0.66443
380	C	Uncoated	0.30250	0.66262
331	C	Uncoated	0.37337	0.66889
285	C	Uncoated	0.46990	0.66481
229	C	Uncoated	0.60046	0.66207
